# Burn Injuries Caused by Hot Water Bottles: Audit and Loop Closure

**Published:** 2010-01-18

**Authors:** Alex Whittam, Ailsa Wilson, John E. Greenwood

**Affiliations:** ^a^University of Leeds Medical School, Leeds, United Kingdom; ^b^Department of Urology, Mater Health Services, Brisbane, Queensland, Australia; ^c^Burns Unit, Royal Adelaide Hospital, Adelaide, South Australia, Australia

## Abstract

**Objectives:** To reduce the annual incidence of hot water bottle burns by reviewing cases, classifying injury, invoking a multimedia campaign to increase public awareness. To stimulate state, federal, and international government review of manufacturing standards with a view to reinforcing legislation. **Methods:** A multimedia warning was launched following the scalding death of an elderly lady. Our database allowed identification of all patients injured by hot water bottles; their medical notes were thoroughly reviewed. **Results:** The review divided cases into bottle failure and/or patient misuse. Early legislation in New Zealand resulted in bottle withdrawal from sale, mirrored by recent legislation in Australia. Legislation and the multimedia campaign have seen admissions from hot water bottle burns markedly decreased. **Conclusions:** Thorough audit of specific burn etiologies, with effective broadcasting of hazard, can result in reduction or abolition of future injury from that source and may prompt appropriate legislation to further protect the general public.

It is estimated that approximately 1% of the population of Australia and New Zealand sustain burn injuries each year.^1^ The Royal Adelaide Hospital is the sole tertiary adult burn centre for South Australia and the Northern Territory, with a 2.4 million km^2^ catchment area populated by approximately 1.7 million people. In 2004, there was a noticeable increase in the number of injuries related to hot water bottles presenting to the burns unit. This culminated in the death of an elderly woman following a hot water bottle burn injury at a nursing home, leading to a coronial inquiry. This case generated considerable public alarm and a demand for information. A retrospective audit was undertaken to evaluate the scale of the problem, and a media campaign (newspaper, radio, and television) was instigated by the 2 medical directors of the adult and pediatric burn services to warn the general public on the potential hazards of hot water bottle use. This was followed immediately by legislative change in New Zealand and more recently a number of legislative changes in Australia, with hundreds of thousands of substandard hot water bottles removed from sale.

Hot water bottles are popular, used throughout much of the world specifically for relief of joint discomfort and menstrual period pain and generally for the initial heating of cold bedsheets. This latter use began in the 16th century, when metal containers, filled with hot coals, were placed into the bed to warm the sheets and removed before retiring (bedpans). Subsequently, safer “bottles,” made of zinc, copper, glass, earthenware, or wood, were introduced and filled with hot water. The design of modern hot water bottles, manufactured in rubber or polyvinyl chloride (PVC), was patented by a Croatian inventor, Eduard Penkala (also the inventor of the mechanical pencil and ink pen and from whose surname the term “pen” derives).^2^

In Australia, it is estimated that 500,000 hot water bottles are sold annually, predominantly imported from China. Rubber hot water bottles are typically cheaper than those of PVC, and thus are estimated to comprise 90% of the market share. The modern hot water bottle consists of 2 main parts: the body/neck, containing the aperture for filling, and a stopper, which tightly seals the bottle. A number of hot water bottles are now sold with a decorative cover, often of a novelty design (eg, cartoon characters) to increase their appeal.^3^

Although relatively simple in design and instructions for use, the benign appearance disguises the danger that individuals can expose themselves to when using (or misusing) the appliance.

A number of reports regarding injury from other heating implements (electric heating pads^4^^-^8 and electric blankets^9^,^10^) have been published, but literature is scant regarding hot water bottles. There are a handful of case reports; a burn in an area of anesthetized skin following breast reconstruction surgery,^11^ perianal burns when used for relief of a painful perianal fissure,^12^ and 2 cases of foot burns in patients with diabetes.^13^ An article documenting the epidemiology of hot water bottle burns treated in the Changhai Hospital Burn Centre in China^14^ presented a considerable case series.

## AIMS

- To describe the demographics and trends seen in patients admitted following hot water bottle burn injuries
- To explain what action was taken in terms of education and legislation to influence subsequent injury
- To evaluate the effect these aforementioned actions had on subsequent burn presentations to the adult tertiary burn service

## METHODS

The South Australian Adult Burns Database has collected information on the mechanism and management of all burns admitted to the Royal Adelaide Hospital Burns Unit since 1998. Patients reporting a hot water bottle as the cause of injury from January 1998 to September 2009 were identified and their medical case notes reviewed retrospectively, paying particular attention to mechanism, treatment, and outcome.

## RESULTS

Twenty-three patients were identified as having had hot water bottle burns since January 1998. Their mean age was 53.2 years (range 17-92 years). Seventeen patients were female. Fourteen patients received hot water scald injuries when the bottle burst or split, or when hot water leaked from the lid or while filling the bottle. Seven received contact burns due to exposure of the skin to the hot surface of the bottle (neuropathic or anesthetic skin or during “assisted” sleep). The mechanism in 2 cases was not recorded.

Four patients were elderly (older than 80 years). Four patients had a diagnosis of diabetes mellitus, of which 2 had been injured as a result of prolonged contact with the hot water bottle in areas of peripheral neuropathy. In the other 2 cases, scalds were ignored because of peripheral neuropathy, resulting in delayed presentation. One postmastectomy patient, who had undergone free flap and implant breast reconstruction 9 years previously, sustained a contact burn in a residual anesthetic area of the reconstructed breast. In 2 cases, alcohol consumption and intoxication accounted for injury: one contact burn from falling asleep on the hot water bottle and the other a scald while attempting to fill the hot water bottle postdrinking. One patient had been critically unwell and undergoing chemotherapy treatment for acute leukemia at the time of injury. He identified heavy sedated sleep as the cause for prolonged contact time with the hot water bottle.

Seventeen patients received burn injuries to the lower limbs, buttocks, or perineum. The remaining areas to be affected were the chest, abdomen, or arms. Mean total body surface area of burn was 3.2%, with the largest being 10%.

Eleven patients required split skin grafting. Of the others, 1 was medically unfit to undergo surgery, 2 had burn excision and primary closure, and 4 received Biobrane.

Length of stay ranged from 1 to 31 days, with a mean of 8.8 days. Longer hospital stay was related to general ill health, exacerbation of preexisting medical conditions, or burns in difficult areas. The fatality was an elderly female patient with preexisting medical problems, who developed sepsis and exacerbation of congestive cardiac failure, dying 14 days after her 7% total body surface area burn. Five patients had delayed presentation with wound infection/cellulitis, while another 3 patients developed infections postoperatively. Among these patients, there were 2 with diabetes who had undergone adequate initial debridement and skin grafting with appropriate antibiotic cover and glycemic control, but who still developed osteomyelitis requiring amputation. Two patients were readmitted due to inadequate social and community resources to enable independent living during the recovery period. Two patients required further reconstructive surgery 1 year postinjury due to hypertrophic scarring and contracture development.

## DISCUSSION

In this, the largest series of hot water bottle burns reviewed outside China, two categories of injuries have been identified; those which occur primarily as a result of misuse or other patient factor and those that occur due to mechanical failure and faulty manufacture of the bottle itself. In a number of cases, injuries led to protracted hospital stay or physical or social complication.

Patient misuse of hot water bottles or ignorance of their correct usage played a role in a number of the burn injuries sustained in the present study. There are a number of recommendations given in regards to the correct and safe usage of hot water bottles (filling with hot water from the tap, not boiling water, filling to a maximum of 3 quarters full, expelling all air above the water level before sealing, etc). One or more of these instructions had been ignored in many of the cases described. In some instances, patients had put a considerable amount of pressure on the hot water bottle, by laying or sitting on it, causing it to burst. Most reputable bottles carry guidelines for safe use, which include avoidance of such practices, some going as far as to suggest removing the bottle from the bed before retiring, ignored by several patients. This practice not only increases potential contact time (compounded by neuropathy or in “assisted” sleep) but also increases the likelihood of pressure rupture.

In those with diabetes, peripheral neuropathy played a significant role in enabling greater contact time, causing significant injury, and in delaying presentation due to an inability to detect the extent of the injury. Patients with diabetes have a greater incidence of wound infection, not only because of delayed presentation but also because the hyperglycemic environment is appreciated by bacteria^15^,^16^ and neuropathic skin is slower to heal. They are also more likely to have coexisting medical comorbidities, thereby increasing the likelihood of complications and their length of stay in hospital,^17^ as was the case in this particular study.

Mechanical failure of the hot water bottle was indicated as a cause for burn injury in at least 10 of the patients. In Australia, it is estimated that 40% (approximately 200,000) of all hot water bottles sold annually are potentially unsafe. The globally accepted standard for the safety of hot water bottles is provided by the British Standard Institute. The current standard is BS 1970:2006 which outlines the minimum specifications for safe hot water bottles, addressing varied parameters from the thickness of the material used and how the product should perform in strength and leakage tests to information/instruction provision and labeling. The British standard is not legislation, but a guideline for the manufacture and control of safe products.

The retrospective nature of this study makes it difficult to distinguish the underlying reasons for the failure of hot water bottles. For example, in the case of rupture, it is impossible to distinguish whether this was due to poor seam strength, degradation of the material, or misuse on the part of the patient. It is probable that some injuries occurred because of interplay of a number of factors. Similarly, when water was spilt during filling, was this due to carelessness on the part of the patient or an inadequately sized aperture? Thus, when attempting to prevent subsequent injury, it is important to adopt a multipronged approach, targeting both mechanical failure issues and consumer misuse of hot water bottles.

Following a series of hot water bottle injuries being admitted to the burns unit of the Royal Adelaide Hospital and the death of one patient, a media campaign was instigated to tackle the problem. Articles appeared in local newspapers in Adelaide and surrounding suburbs, messages appeared during ABC radio news bulletins, and a piece appeared on an ABC television news program.

Following the initial media releases, changes began to take place in national and international legislation. In November 2004, the Minister of Consumer Affairs in New Zealand declared that *rubber* hot water bottles that did not meet BS 1970:2001 standards were to be declared unsafe, thus removing a great number from the market under the Fair Trading Act 1986. This declaration was not permanent legislation but remained in effect for 18 months. During this period, the issue was reexamined in greater detail. As part of the consultation process by which permanent laws are established, a discussion document released in October 2005 summarized a range of information concerning hot water bottles. This discussion made reference to the death at the Royal Adelaide Hospital as a result of a hot water bottle burn.^18^ The ban was extended for a further 12 months, and in 2007, an unsafe goods (hot water bottles) notice was issued prohibiting the supply of *rubber* and *PVC* hot water bottles for a further 12 months. To supply or import rubber or PVC hot water bottles, a certificate must be held which shows the product complies with BS 1970:2001 or BS 1970:2006 standards and has been tested in an accredited laboratory.^19^,^20^ This became an indefinite ban under the Fair Trading Act 1986 in December 2008.^21^

In Australia, there has been statewide legislation banning the supply of hot water bottles that did not comply with BS 1970:2001 standards in place in Victoria^22^ since 2005. In 2007, the Australian Competition and Consumer Commission released a document discussing possible legislation changes to regulate hot water bottles under the Trade Practices Act 1974, again making reference to the death of the elderly female patient at the Royal Adelaide Hospital. In 2008, there was a Commonwealth (federal) legislative amendment to the Trade Practices Act 1974, regulating the manufacture of rubber and PVC hot water bottles and describing the minimum safety requirements in line with BS 1970:2006 standards.^23^ Following this change in Commonwealth law, the various states in Australia amended their own individual laws accordingly.

There have been a number of press releases concerning hot water bottle safety, the most recent by the Australian Competition and Consumer Commission in September 2009. This media alert stated that of 30 brands of bottle tested, 18 did not comply with current safety standards, leading to a recall of 250,000 products. Consumers were also advised to discard any hot water bottles that were older than 1 year.^24^ Media releases from other Australian states, such as Victoria, have also carried similar messages and have informed the public of changes in legislation.^25^

Unfortunately, media campaign messages tend to be generally short-lived and quickly forgotten. Regular public addresses (possibly television community service announcements) may be necessary to reiterate important safety information. The changes in legislation may not be completely effective in removing dangerous products from the environment since many of the regulations came into effect only recently; logically, there will be a number of hot water bottles purchased before the laws came into effect. The instruction issued to dispose of devices older than 1 year should help combat this. Some reports suggest that the BS 1970 safety standards are not sufficiently rigorous or demanding. One such report was issued by a rubber and plastic consultancy that tested a hot water bottle that conformed to BS 1970 standards and which had failed after 3 months' use. The report suggests that the BS 1970 safety standards need to be refined.^26^

## CONCLUSIONS

A multifaceted campaign was implemented to alert people to the danger of injuries from hot water bottles. A media campaign aiming to reach as many people as possible was instigated in Adelaide, using the printed press, radio, and television outlets. The publicity generated from this and the death of an elderly patient following a hot water bottle burn led to changes in legislation, first in New Zealand and later in Australia. This focused campaign has had a noticeable effect on admissions from these injuries to the burns unit at the Royal Adelaide Hospital. From January 2004 to December 2005, there were 10 injuries which warranted admission. Since the media campaign and changes in legislation in Australia came into effect, from January 2008 to September 2009, there have been only 2 admissions following hot water bottle burns.
